# Seasonal Dynamics of Sediment Microbial Communities at Different Distances from Artificial Reef Units

**DOI:** 10.3390/microorganisms13061194

**Published:** 2025-05-23

**Authors:** Meiling Zhu, Yanli Tang

**Affiliations:** College of Fisheries, Ocean University of China, Qingdao 266003, China; zml5660@stu.ouc.edu.cn

**Keywords:** sediment microorganisms, artificial reef units, spatio-temporal characteristics of community, reef distance

## Abstract

Artificial reefs (ARs) are important for habitat restoration and exhibit clear spatial gradient effects. However, most studies focus on AR groups, neglecting the ecological functions and mechanisms of individual AR units at a local scale. This limits a deeper understanding and optimization of the ecological effects of ARs. This study employed high-throughput sequencing to examine the seasonal and spatial variations in sediment microbial communities surrounding AR units. The results showed that microbial community distributions in sediments varied significantly with seasons, reef distance, and reef structure. The community structure varied significantly across seasons at different reef distances, with the most unique structure observed at 3 m (1.5-times the reef height). In May, microbial community spatial changes were mainly driven by sediment organic matter (OM), while in November and February, although the spatial enrichment patterns of microbial groups changed seasonally, they were not strongly affected by functional types (aerobic or anaerobic). The Neutral Community Model (NCM) showed lower levels of R^2^ and Nm at 0 m and 3 m, which are relatively consistent with the flow field effects (upwelling and reverse eddy currents). Key environmental factors and their regulatory effects vary with distance from the reef.

## 1. Introduction

Artificial reef (AR) changes typically occur in the surrounding benthic organisms, sediment characteristics, hydrodynamic environment, and biological community structure, forming a special ecological ring [1]. Studies suggest that ARs effectively attract fish and increase local population density at various spatial scales [2,3,4,5]. Reeds et al. [5] found that the effective ecological area around an AR unit could extend to 15-times its physical area. Benthic communities near reefs often show increases in abundance or biomass [5,6,7]. However, this ecological effect is not always one-way. The increase in fish and large benthic animals may reduce the abundance of some prey species [8,9] and, in some cases, lead to competition or redistribution of local resources. Additionally, feeding, excretion, and other disturbances by organisms around the reef further enhance the spatial ecological effects [10,11].

The ecological role of ARs is not only reflected in the restructuring of biological communities, but their hydrodynamic effects are also an important mechanism influencing benthic communities. The upwelling in front of the reef and the vortex flow in the wake area are key hydrodynamic features of ARs [12]. Upwelling transports nutrients to the upper layers, providing abundant resources for plankton and filter-feeding organisms, while the vortex shedding in the wake area offers refuge for marine life to reproduce and settle [13]. Furthermore, the hydrodynamic effects also influence sediment distribution, nutrient transport, and particle settling [12,14,15], which in turn impact the spatial distribution and ecological function of organisms. The flow field effect of the AR unit is influenced by factors such as reef size, shape, depth, and flow speed [16,17]. Kim et al. [18] found a strong linear relationship between the wake length and reef height, with the proportional coefficient ranging from 0.27 to 3.03, showing no correlation with flow speed.

Sediment microbial communities are an important part of sediment habitats, characterized by high inherent specificity and sensitivity to biogeochemical changes [19,20,21,22]. The biological community restructuring and hydrodynamic changes caused by ARs can directly or indirectly drive responses in sediment microbial community structure and function. Most studies focus on the overall ecological effects of ARs at the community level, with fewer studies on the local ecological effects around single AR unit. This study investigates the spatial ecological effects of microbial communities in sediment around the AR unit, helping to reveal the impact mechanisms on the local sediment environment and the formation of microscale ecological effects.

The study employed high-throughput sequencing to analyze the seasonal dynamics of sediment microbial communities at varying distances from AR units (0 m, 1 m, 3 m, and 5 m). The main objectives are (1) to clarify the impact of the AR unit on the composition and structure of sediment microbial communities at different reef distances, (2) to explore the effects of the reef unit on the spatial distribution and ecological functions of surrounding sediment microbial communities, and (3) to investigate the key environmental drivers shaping microbial community structure at different reef distances.

## 2. Materials and Methods

### 2.1. Study Area and Sample Collection

Laoshan Bay is located in the eastern part of Laoshan, with an area of approximately 188 km^2^. It is a typical semi-enclosed bay characterized by reciprocating tidal currents, with flow directions approximately perpendicular to the coastline. The construction of ARs was completed in 2018, covering approximately 210,000 m^2^, with an average water depth of around 10 m. The AR units are arranged in spaced rows parallel to the shoreline, with reef dimensions of 2 m × 2 m × 2 m.

**Figure 1:**
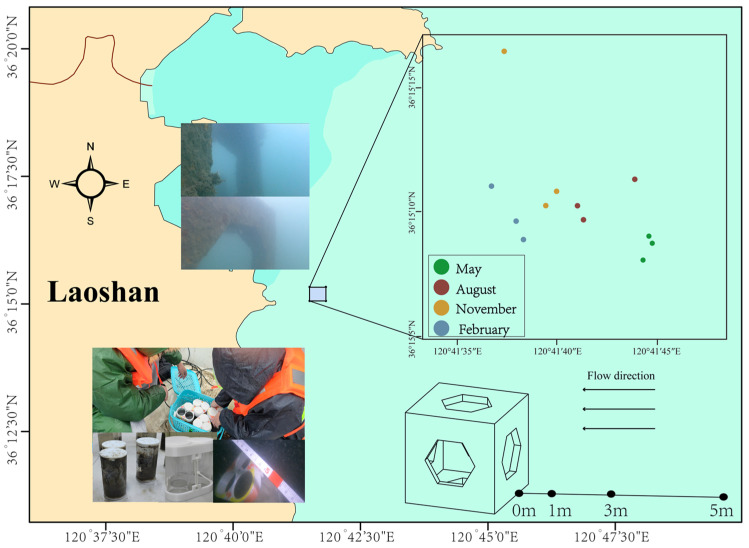
Sampling stations of artificial reef units in Laoshan Bay.

**Figure A1:**
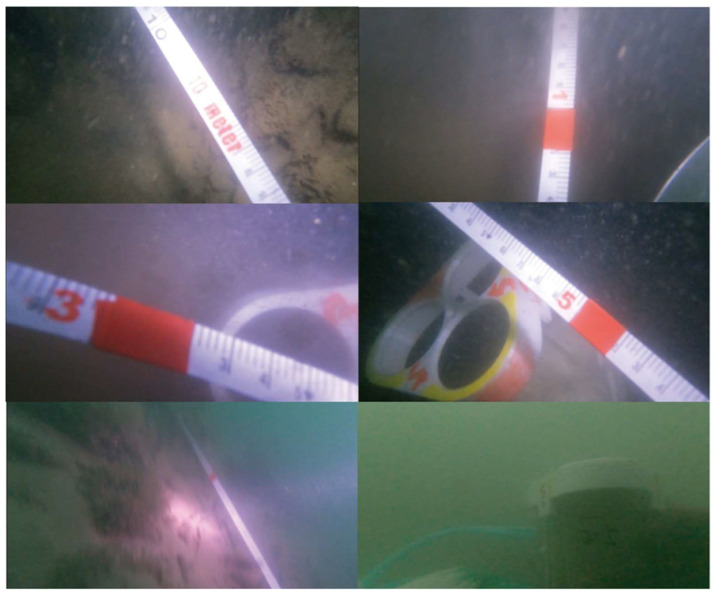
Fixed interval sampling method.

In May, August, and November of 2023, and February of the following year, sediment samples were collected around 12 AR units. Divers selected three random reef units per quarter for sampling (Figure 1). Sediment samples were collected at 0 m, 1 m, 3 m, and 5 m from the reef along the flow direction, using measuring tapes. During sampling, the centerline of the reef’s upstream face was designated as the 0 m point. Surface sediment samples (0–5 cm) were then collected at distances of 0 m, 1 m, 3 m, and 5 m from the reef using a 6 cm diameter coring tube (Ruici, Anhui, China) (Figure A1). Three replicate samples were taken at each distance, and an underwater video was recorded at the same time.

After sampling, all samples were immediately placed in ice boxes and transported to the laboratory within 2 h. The sediment samples were divided into two parts: one was stored at −80 °C for microbial sequencing analysis, and the other was kept at −20 °C for environmental parameter measurement.

### 2.2. Physicochemical Analyses

For the sediment samples, sediment bulk density (BD) was determined by dividing oven-dry mass obtained after drying at 105 °C for 72 h by the volume of the sediment. Water content (WC) was obtained by measuring the weight loss after the sediment was dried at 70 °C for 72 h until a constant weight was attained. Organic matter content (OM) was measured by burning sediment to ash at 550 °C for 4 h. The sediment pH and electrical conductivity (EC) were measured in a 1:2.5 (*w*/*v*) mixture of sediment and deionized water. Sediment nitrate-N (NO_3_^−^-N), ammonium-N (NH_4_^+^-N), available sulfur (AS), total phosphorus (TP), inorganic phosphorus (IP), and organic phosphorus (OP) were quantified using corresponding detection kits (Solarbio, Beijing, China). Additional details are provided in the Appendix A.

### 2.3. DNA Extraction, PCR Amplification, and High-Throughput Sequencing

Microbial DNA was extracted from sediment samples using the E.Z.N.A.^®^ Soil DNA Kit (Omega Bio-tek, Norcross, GA, USA), according to manufacturer’s protocols. The V3-V4 region of the bacteria 16S ribosomal RNA gene was amplified by PCR (95 °C for 2 min, followed by 25 cycles at 95 °C for 30 s, 55 °C for 30 s, and 72 °C for 30 s and a final extension at 72 °C for 5 min) using primers 338F (5′-ACTCCTACGGGAGGCAGCAG-3′) and 806R (5′-GGACTACHVGGGTWTCTAAT-3′), where the barcode is an eight-base sequence unique to each sample. PCR reactions were performed in triplicate with a 20 μL mixture containing 4 μL of 5 × FastPfu Buffer, 2 μL of 2.5 mM dNTPs, 0.8 μL of each primer (5 μM), 0.4 μL of FastPfu Polymerase, and 10 ng of template DNA. Amplicons were extracted from 2% agarose gels and purified using the AxyPrep DNA Gel Extraction Kit (Axygen Biosciences, Union City, CA, USA), according to the manufacturer’s instructions. Paired-end sequencing of amplicons was performed on an Illumina Mieseq platform (PE250 mode) (Shanghai Biozeron Biotech, Shanghai, China). Raw sequences were quality filtered and assembled. The DADA2 algorithm in Quantitative Insights Into Microbial Ecology (QIIME, V1.9.0, http://qiime.org/scripts/assign_taxonomy.html, accessed on 4 July 2024) was used to identify indel mutations and substitutions in the merged sequences. The paired reads were trimmed and filtered with a maximum of two expected errors per read (maxEE = 2). After paired sequences were merged and chimeras were filtered, amplicon sequence variants (ASVs) were generated. The phylogenetic affiliation of each 16S rRNA sequence was identified by the RDP Classifier (http://rdp.cme.msu.edu/, accessed on 4 July 2024) against the Silva (SSU138) database using a confidence threshold of 70%. The raw reads were deposited into the NCBI Sequence Read Archive (SRA) database (Accession Number: PRJNA1197441).

### 2.4. Statistical Analyses

Principal Coordinate Analysis (PCoA) based on the Bray–Curtis distance matrix was performed using the “ape” package (v5.4-1) to analyze differences in microbial community composition. Permutational Multivariate Analysis of Variance (PERMANOVA) and Analysis of Similarity (ANOSIM) were used to investigate the spatiotemporal effects on microbial communities. The Kruskal–Wallis test was applied to analyze statistical differences, with significance set at a *p*-value of 0.05. Venn diagrams from the “VennDiagram” package (v1.6.20) were used to analyze ASV composition differences. Additionally, the Linear Discriminant Analysis Effect Size (LDA) method (LEfSe) was employed to identify significant biomarkers between groups using the “indicspecies” package (v1.7.12) [23]. The ABT model and Spearman correlation, implemented via the “gbm” package (v2.1.8) and “linkET” packages (v0.0.6), explored associations between environmental factors and microbial communities. Statistical analyses were conducted in R 4.0.2. Environmental factors were presented as mean ± standard error (SE) in Excel 2016 (Microsoft Office 2016, Microsoft, Redmond, WA, USA). The Neutral Community Model (NCM) was further used to evaluate the contribution of random processes to microbial community assembly by predicting the relationship between the occurrence frequency and relative abundance of each ASV [24], with the analysis conducted on the Tutools platform (https://www.cloudtutu.com, accessed on 2 March 2025).

## 3. Results

### 3.1. Microbial Community Composition

**Figure A2:**
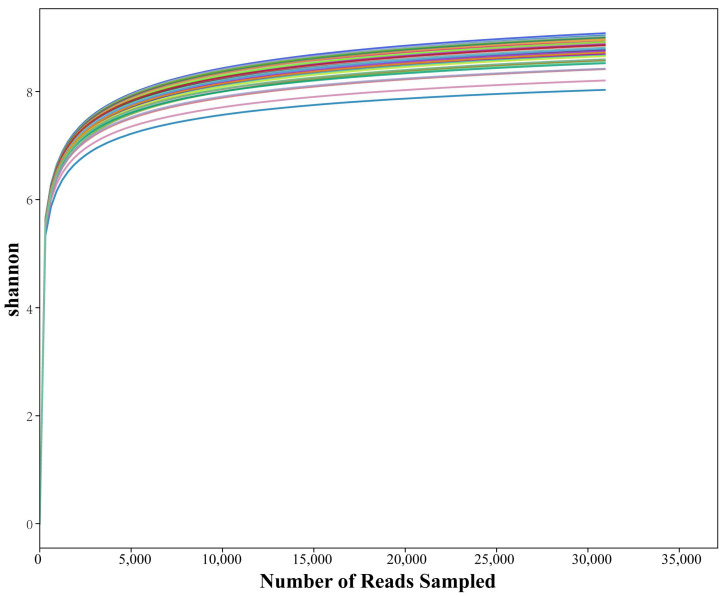
Shannon–Wiener curves for each sample. Colored curves indicate different samples.

A total of 498,076 ASVs were detected across 48 samples, obtaining a total of 3,054,574 16S rRNA gene sequences. The highest number of ASVs was recorded in May (131,076), followed by August (126,759) and November (125,076), with the lowest in February (115,165). Rarefaction curves for all samples plateaued, indicating comprehensive representation and identification of bacterial communities (Figure A2).

**Figure 2:**
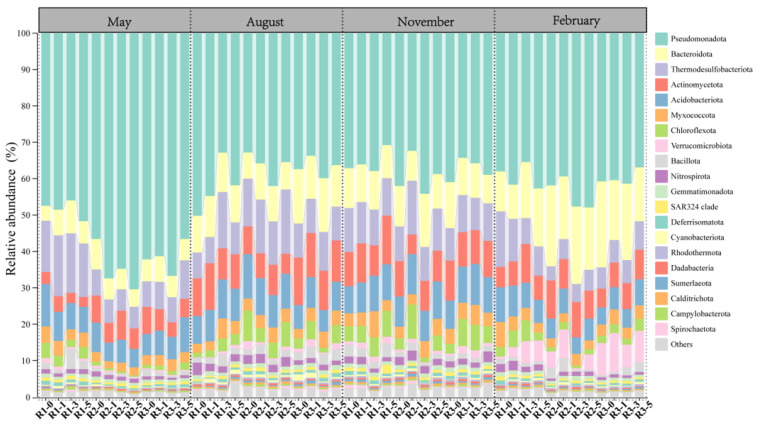
Relative abundance of bacterial communities at the phylum level at different distances from the AR units.

The microbial community structure showed notable seasonal and spatial fluctuations, particularly among dominant phyla with relative abundances above 1% (Figure 2). Significant differences with distance from reefs were observed for microbial communities at all three reefs in May (involving 12 phyla) and February (involving 11 phyla). In contrast, only two reefs exhibited significant differences in August, and none were detected in November (Table A1). Some phyla displayed dynamic spatial patterns across seasons; for instance, the relative abundance of Actinomycetota sharply decreased at 0 m in February but peaked at this distance in May. Myxococcota abundance increased initially with distance before declining in May and August, while it steadily declined in February. Verrucomicrobiota abundance decreased with distance from reefs in May but showed the opposite trend in February.

Certain phyla maintained consistent spatial patterns across seasons (Table A1). Bacteroidota abundances consistently increased with greater distance from reefs in both May and February. Similarly, Thermodesulfobacteriota were significantly enriched with increasing distance in May, August, and February. Acidobacteriota showed seasonal stability, consistently peaking at the 0 m distance in May, August, and February.

Additionally, microbial distribution patterns varied notably among different reefs (Table A1). For example, Acidobacteriota, Thermodesulfobacteriota, Chloroflexota, and Myxococcota significantly increased at 3 m from reef R1 in August but decreased at reef R2. In contrast, dominant phyla in May and August often reached maximum or minimum abundances at distances of 0 m and 5 m.

### 3.2. Spatio-Temporal Dynamics of Microbial Community Structure

**Figure 3:**
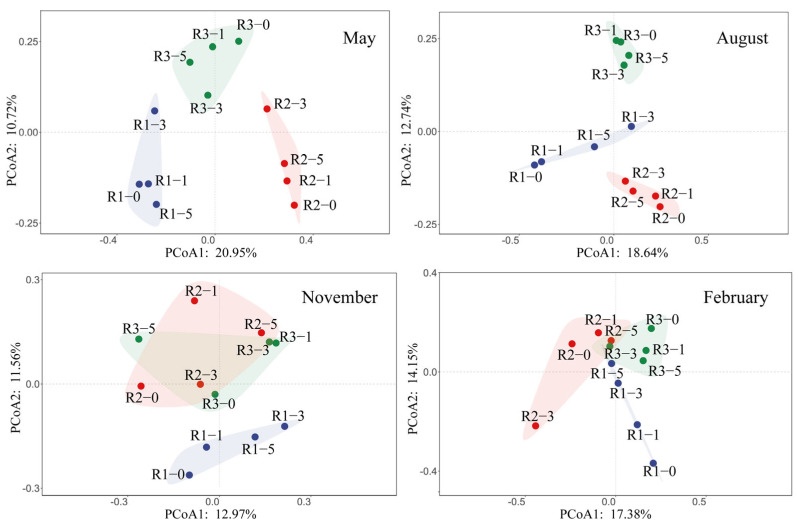
Principal coordinates analysis (PCoA) plots of bacteria across four seasons in three AR units.

Principal coordinate analysis (PCoA) indicated significant seasonal and spatial effects on microbial communities in sediments around artificial reefs (Figure 3). Community structures exhibited distinct seasonal patterns, with the greatest dispersion observed in August and relative clustering in February. In May and February, sediment microbial communities at 0 m, 1 m, and 5 m distances showed higher similarity, whereas communities at 3 m were notably distinct. In August, however, microbial communities at 0 m and 1 m were similar, as were communities at 3 m and 5 m. In November, community structures largely overlapped across different reefs, suggesting minimal spatial differentiation.

**Figure 4:**
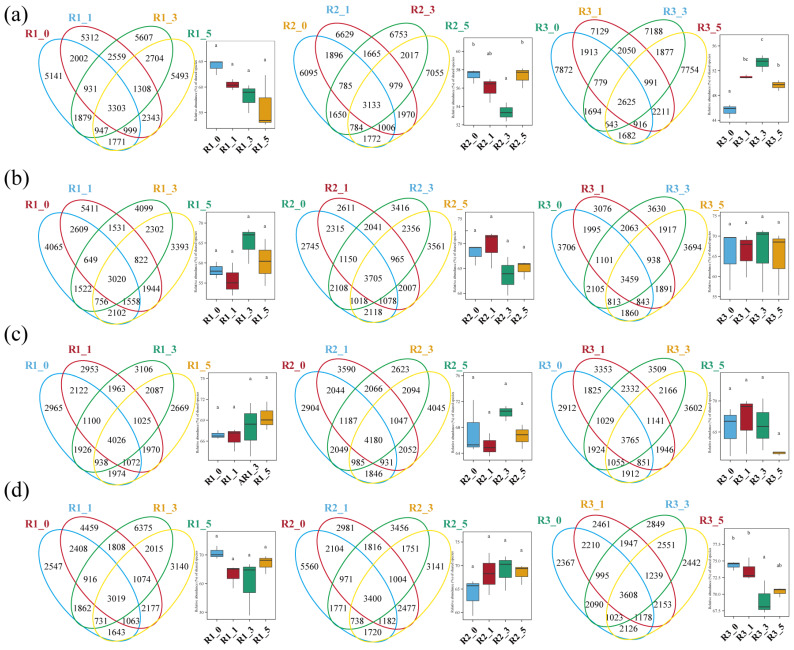
Venn diagrams of core ASVs in three AR units across May (**a**), August (**b**), November (**c**), and February (**d**), with bar plots showing intergroup differences among reef distances. Means with the same letter are not significantly different, while those with different letters indicate a significant difference (*p* < 0.05).

Analysis of shared and unique ASVs showed that May had the highest number of unique ASVs (ranging from 5141 to 7872). The lowest numbers of shared ASVs between 0 m and 1 m samples were observed in May and February, whereas November had the greatest number of core ASVs across all distances. In August, the number of core ASVs at 0 m and 1 m was similar, as were those at 3 m and 5 m, consistent with the PCoA results. Additionally, in May, core ASV counts at 3 m from reefs R2 and R3 showed substantial variation. Overall, shared ASVs exhibited no clear spatial gradient across the four distances (Figure 4).

### 3.3. Differences in Microbial Structure at Various Reef Distances

**Figure 5:**
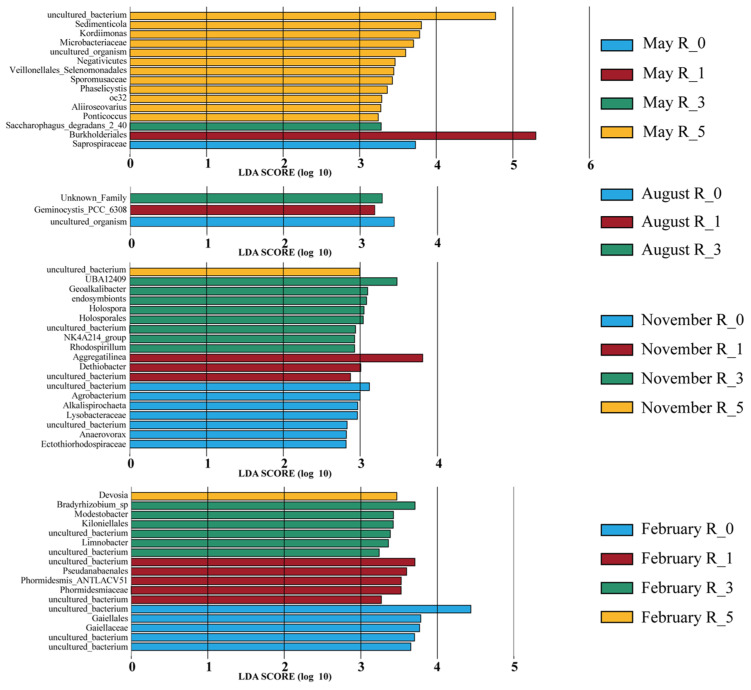
Indicator species of microbial communities in sediment at different distances from the AR units (LDA = 2).

LEfSe analysis identified significant microbial taxa differentiating distances from reefs across seasons. November exhibited the highest number of differentiating clades (19), predominantly at the family level (Figure 5). In contrast, August had the fewest differentiating taxa (only three). In May, among the 15 clades detected, 12 clades were significantly enriched at 5 m, including 1 class (Negativicutes), 1 order (Veillonellales_Selenomonadales), 2 families (Sporomusaceae, Microbacteriaceae), and 6 genera (*Kordiimonas, Sedimenticola, oc32, Aliiroseovarius, Ponticoccus, Phaselicystis*), as well as 2 uncultured_bacterium (*Mesorhizobium*, *YC_ZSS_LKJ63*). Single clades were identified at 0 m (family Saprospiraceae), 1 m (order Burkholderiales), and 3 m (species *Saccharophagus degradans*). In November, eight taxa were enriched at 3 m, including one order (Holosporales), six genera (*UBA12409, Geoalkalibacter, endosymbionts, Holospora, NK4A214_group, and Rhodospirillum*), and one species (*NK4A214_group*). At 0 m, seven taxa were enriched, comprising two families (Lysobacteraceae, Ectothiorhodospiraceae), three genera (*Agrobacterium, Alkalispirochaeta, Anaerovorax*), and two species (*Dasania*, *Anaerovorax*). At 1 m, two genera (*Aggregatilinea, Dethiobacter*) and one species (*Desulfatirhabdium*) were significantly enriched, while only one species (*Desulfuromusa*) was significantly enriched at 5 m. In February, 17 differentiating taxa were detected, distributed fairly evenly at 0 m (order Gaiellales, family Gaiellaceae, species *CSP1_2, Gaiella, and Bauldia*), 1 m (order Pseudanabaenales, family Phormidesmiaceae, genus *Phormidesmis*, species *Synechococcus_PCC_7502* and *Leptolinea*), and 3 m (order Kiloniellales, genera *Modestobacter* and *Limnobacter*, species *Bradyrhizobium_sp, Limnobacter and Sneathiella*), with only 1 genus (*Devosia*) detected at 5 m.

### 3.4. Community Assembly Mechanisms of Microbes at Varying Reef Distances

**Figure 6:**
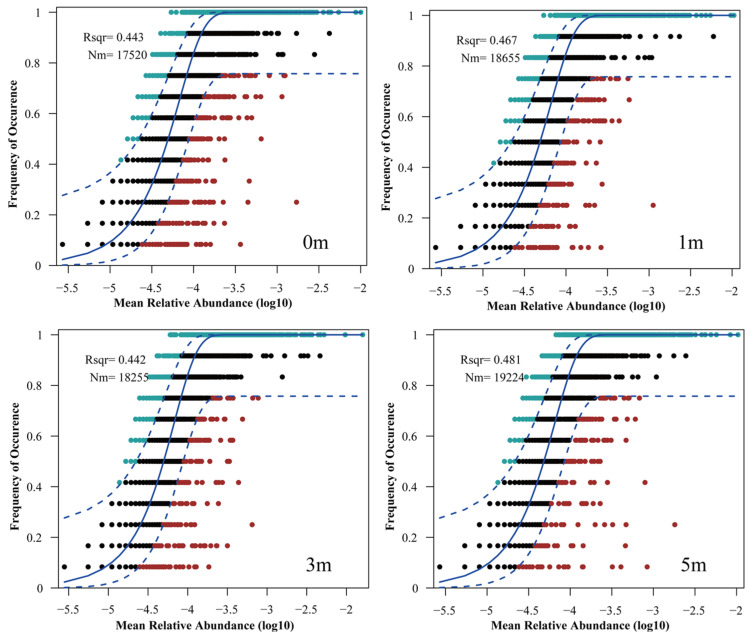
Neutral community model (NCM) fit to microbial community ASV data at different distances from the artificial reef. The solid line represents the optimal fit for the NCM, while the dashed lines indicate the 95% confidence interval around the model predictions. Rsqr (R^2^) indicates the fit of the model, Nm represents the product of metacommunity size and migration, and m indicates the estimated migration rate.

The Neutral Community Model (NCM) explains microbial community composition and dynamics, offering insights into assembly mechanisms. The NCM explained a substantial portion of community variance: 44.3% at 0 m, 46.7% at 1 m, 44.2% at 3 m, and 48.1% at 5 m (Figure 6). The highest explained variance and Nm (migration) value (19,224) were at 5 m, while 0 m and 3 m showed lower values.

The model also estimates the Nm value, which quantifies microbial dispersal and migration patterns under different conditions. The results showed the highest Nm value at 5 m (19,224), indicating the strongest dispersal, followed by 1 m (18,655), which was similar to 3 m (18,255), and the lowest at 0 m.

### 3.5. Relationship Between Dominant Phyla and Environmental Factors

**Figure 7:**
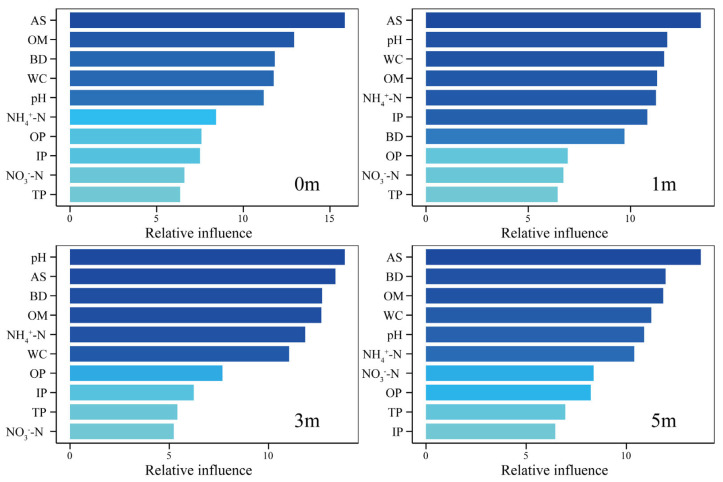
Identification of key environmental factors for ABT in 0 m, 1 m, 3 m, and 5 m. The height and color of the bars represent the proportion of community variation explained by each environmental factor. NH_4_^+^-N—ammoniacal nitrogen; NO_3_^−^N—nitrate nitrogen; AS—available sulfur; IP—inorganic phosphorus; TP—total phosphorus; OP—organic phosphorus; WC—water content; BD—bulk density; OM—organic matter content.

Key environmental factors influencing sediment microbial community structure show dynamic adjustments with distance from the reef. Additionally, the distribution trends of different sediment environmental factors vary seasonally across reef distances (Figure 7, Table A2). The study found that active sulfur (AS), organic matter (OM), water content (WC), and pH are significant drivers of microbial community structure at distances of 0 m, 1 m, 3 m, and 5 m, with their contributions to community variation exceeding 10% (Figure 7). Among these, AS remained relatively stable across distances, maintaining high contribution levels. OM had its highest contribution at 0 m (12.93%) and 3 m (12.66%), while pH increased initially and then decreased with distance, peaking at 3 m (13.84%). Ammonium nitrogen (NH_4_^+^-N) followed a similar trend, with its maximum contribution at 3 m and minimum at 0 m (8.43%). WC showed an initial decrease and subsequent increase with distance, peaking at 0 m and reaching its lowest point at 3 m. Bulk density (BD) contributed over 10% at 0 m, 3 m, and 5 m, but only 9.70% at 1 m. Inorganic phosphorus (IP) exceeded 10% contribution only at 1 m.

**Figure 8:**
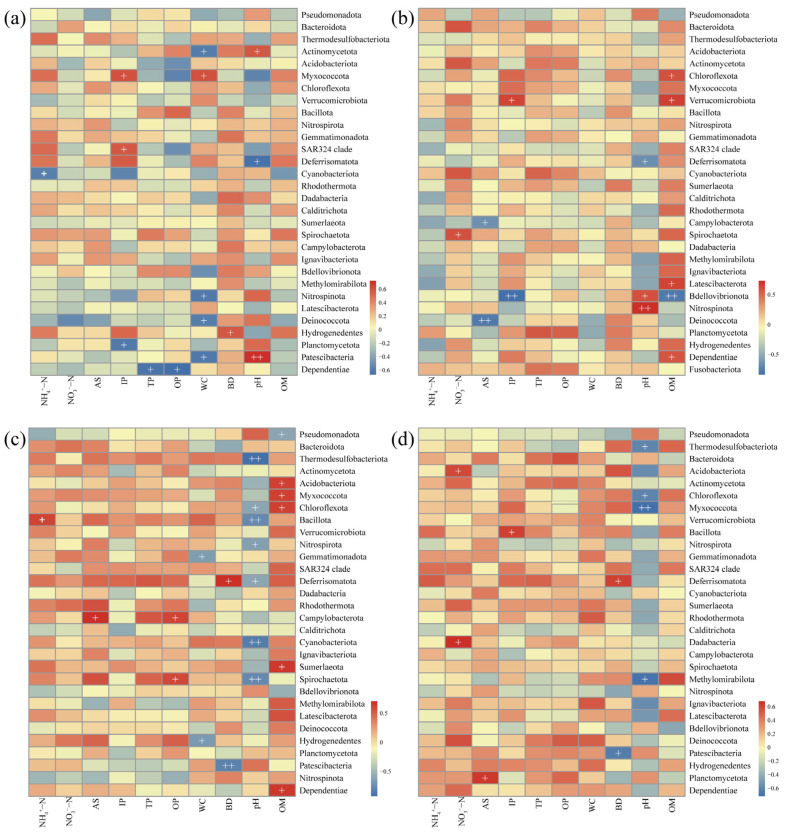
Correlation analysis between abundant microbial taxa (phylum) and environmental factors at different reef distances: (**a**) 0 m, (**b**) 1 m, (**c**) 3 m, and (**d**) 5 m. NH_4_^+^-N—ammoniacal nitrogen; NO_3_^−^-N—nitrate nitrogen; AS—available sulfur; IP—inorganic phosphorus; TP—total phosphorus; OP—organic phosphorus; WC—water content; BD—bulk density; OM—organic matter content.

To better understand the effects of AR units on surrounding sediment microbial communities, we analyzed the correlation between the relative abundance of the top 30 microbial phyla and environmental factors at different reef distances (0 m, 1 m, 3 m, 5 m). The results revealed dynamic changes in the correlation between dominant microbial phyla and environmental factors with distance, with varying degrees of influence across distances (Figure 8). OM showed no significant correlation with dominant phyla at 0 m and 5 m, but at 1 m, it was positively correlated with Chloroflexota, Verrucomicrobiota, Latescibacterota, and Dependentiae. At 3 m, it was positively correlated with Acidobacteriota, Myxococcota, Chloroflexota, Sumerlaeota, and Dependentiae. Similarly, pH also varied in its regulatory effect with distance, being positively correlated with two dominant phyla at 0 m and 1 m, and negatively correlated with seven phyla at 3 m, including Thermodesulfobacteriota, Chloroflexota, Bacillota, Nitrospirota, Deferrisomatota, Cyanobacteriota, and Spirochaetota. At 5 m, pH was negatively correlated with only four phyla. Physical sediment factors (WC and BD) also showed significant spatial variation in their impact on microbial communities. At 0 m, WC was significantly correlated with Actinomycetota, Myxococcota, Nitrospirota, Deinococcota, and Patescibacteria, while at 3 m, it correlated significantly with Gemmatimonadota and Hydrogenedentes. BD was positively correlated with Deferrisomatota at 3 m and 5 m but negatively with Patescibacteria. Different forms of phosphorus (IP, TP, OP) had varying impacts at different distances, with IP being significantly correlated with three, two, and one dominant phyla at 0 m, 1 m, and 5 m, respectively. TP and OP at 0 m were only negatively correlated with Dependentiae, while OP at 3 m was positively correlated with Campylobacterota and Spirochaetota.

## 4. Discussion

The structure and function of marine sediment microbial communities are significantly regulated by environmental factors, including hydrodynamics, nutrient concentrations, organic matter content, and pH, among other complex elements [25,26,27,28,29]. The deployment of artificial reefs alters local hydrodynamic conditions and promotes the sedimentation of organic matter and nutrients, potentially affecting the composition and metabolic functions of sediment microbial communities [13,30]. However, systematic studies on how microbial communities at different distances from the reef respond to these environmental changes are still lacking. Furthermore, microbial community responses to AR units not only exhibit spatial heterogeneity but are also influenced by seasonal variations. Thus, exploring the spatio-temporal dynamics of sediment microbial communities around AR units is crucial for understanding their ecological functions.

### 4.1. Composition and Seasonal Variation in Sediment Microbial Communities at Different Reef Distances

As a key regulator in marine ecosystems, artificial reefs influence the distribution of benthic organisms and play a significant role in shaping the composition of sediment microbial communities. The impact of artificial reefs on microbial communities primarily occurs through physical environmental changes and ecological process regulation. First, AR units alter hydrodynamic conditions in the sediment environment, forming upwelling zones in front of the reef and low-velocity eddy zones behind it [31]. These changes result in variations in sediment settling rates and physicochemical properties across different areas [30,32]. These hydrodynamic variations not only affect nutrient diffusion but may also shape microbial community niches by altering the patterns of oxygen and organic matter supply [33,34]. Moreover, AR units may promote the enrichment of specific functional microbial groups, such as carbohydrate-degrading bacteria, sulfur-oxidizing bacteria, and nitrogen-cycling microorganisms. The results suggest that microbial community composition at different reef distances follows specific distribution patterns, particularly for dominant phyla with relative abundances exceeding 1%. Some phyla exhibit consistent spatial distribution patterns across seasons. For example, the relative abundance of Thermodesulfobacteriota and Bacteroidota increases with distance from the reef in different seasons. Thermodesulfobacteriota, a key player in sulfate reduction, plays a central role in sulfur cycling in sediments [35]. The increase in their abundance accelerates the sulfur cycle in sediments. Bacteroidota, a major group of carbohydrate-degrading bacteria, proliferates more in environments with higher organic matter content [36,37]. Acidobacteriota, which are oligotrophic and mostly aerobic, are abundant in low organic carbon environments [38]. The decrease in Bacteroidota and the increase in Acidobacteriota indicate a reduced organic matter decomposition rate in microbial communities at 0 m.

However, the spatial distribution patterns of certain microbial phyla at different distances from the reef varied seasonally. For example, members of the phylum Myxococcota are aerobic bacteria characterized by unique metabolic and structural features [39]. Most species within this phylum exhibit independent motility [40] as well as social motility [41]. Their enrichment patterns at different reef distances may reflect habitat selection for suitable environmental conditions. Similarly, Actinomycetota and Verrucomicrobiota, which play crucial roles in the mineralization and remineralization of complex polysaccharides [42,43,44], showed spatial variations closely associated with organic matter distribution.

Additionally, seasonal changes significantly affect the composition and abundance of sediment microbial communities, especially in May and February, where microbial distribution patterns exhibit more pronounced changes. In contrast, microbial communities in August showed more stability, while November exhibited a more homogenized microbial composition, with no significant distance effects detected. Moreover, in May and February, all reefs displayed trends in microbial abundance changes with distance, whereas only some reefs in August showed significant changes, and no clear differences were observed in November. These findings suggest that seasonal environmental factors may modulate the impact of AR units on microbial communities to some extent. These results indicate that the role of AR units in regulating microbial community composition exhibits seasonal variations, but some microbial communities are likely influenced by more stable ecological factors, such as organic matter deposition or hydrodynamic conditions.

### 4.2. Impact of AR Units on Microbial Community Structure and Species Distribution Patterns

The deployment of artificial reefs triggers a series of “ecological ring” effects in marine ecosystems, most notably the attraction of fish and benthic organisms, forming local ecological cores near the reef [5,7]. However, compared to the aggregation effects of larger organisms, microbial community responses are more complex and exhibit unique spatial differentiation. PCoA analysis indicated that microbial community structures at 3 m from the reef in May and February deviated significantly from other areas (Figure 3). This suggests that sediment microbial communities around reefs are less influenced by biological aggregation effects and that spatial variations are mainly regulated by hydrodynamic features and sedimentary environments. Upwelling in front of the reef transports organic matter and nutrients to the upper layers of water, while the eddy flow behind the reef reduces water velocity, promoting the settling of suspended particles [45]. However, the intensity and impact range of upwelling and eddy flow are regulated by factors such as reef type, size, flow direction, and current velocity [12]. Therefore, AR units may not only alter hydrodynamic features but also shape differentiated sediment environments, which likely contributes to the unique microbial community structure at 3 m from the reef.

To further clarify the impact of AR units on sediment microbial communities at various distances, this study applied LEfSe analysis to systematically examine community structural differences and the enrichment of key taxa. The results revealed significant variations in the differential abundance of microbial taxa across seasons and reef distances. In November and February, differential taxa were most significantly enriched at a distance of 3 m from the reef. In May, microbial communities at 5 m showed the greatest enrichment, dominated by anaerobic bacteria and marine heterotrophic bacteria. Specifically, taxa such as Negativicutes, Veillonellales_Selenomonadales, and Sporomusaceae, typical anaerobic bacteria known for their adaptation to low-oxygen conditions [46,47], were highly enriched at this distance. In addition, *Kordiimonas*, *Ponticoccus*, *Phaselicystis*, and *Aliiroseovarius*, important marine heterotrophic genera capable of degrading complex organic compounds and accelerating sediment organic matter decomposition [48,49,50], were also significantly enriched. The enrichment of Saprospiraceae and *Saccharophagus degradans* at 0 m and 3 m, respectively, was closely associated with complex organic matter decomposition [51,52]. These findings indicate that sediment microbial community distribution in spring is primarily driven by sediment organic matter content, with enrichment at 5 m closely related to higher organic matter levels (Table A2). In November, microbial enrichment patterns changed, with significant anaerobic characteristics at distances of 0 m and 3 m from the reef. At the 0 m, four enriched taxa—*Spirochaeta*, *Alkalispirochaeta*, Ectothiorhodospiraceae, and *Anaerovorax*—were identified as anaerobic bacteria [53,54,55,56]. Similarly, at 3 m, five enriched taxa were classified as anaerobes [57,58,59]. Furthermore, anaerobic taxa were also enriched at 1 m and 5 m distances [60,61,62,63]. This trend indicates that the autumn enrichment patterns of anaerobic bacteria were consistent with the overall spatial differentiation, showing the greatest enrichment at 3 m.

In February, microbial enrichment patterns were relatively stable, with taxa distributed evenly across 0 m, 1 m, and 3 m sites, yet displaying functional differences. At 0 m, four aerobic taxa were identified [64,65], whereas at 3 m, five aerobic taxa were observed [66]. At the 1 m site, four taxa were identified as phototrophic bacteria [67]. These results indicate that despite seasonal variations in microbial functional groups (aerobic or anaerobic), their spatial enrichment patterns remained largely consistent. Therefore, AR units shape the spatial distribution of sediment microbial communities without fundamentally altering bacterial ecological characteristics, providing locally favorable conditions for diverse microbial groups.

### 4.3. Microbial Community Assembly Mechanisms at Different Reef Distances

**Figure 9:**
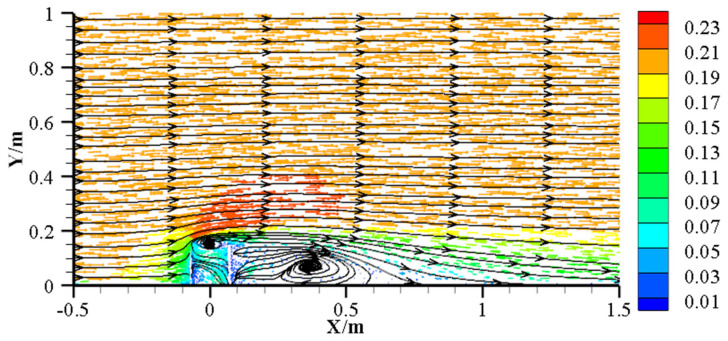
Upwelling and backward eddies around AR units in the central plane, z = 0 [69]. Arrows indicate the direction of water flow, and the color gradient represents flow velocity, with red indicating higher speeds.

To investigate microbial community assembly around AR units, this study employed the Neutral Community Model (NCM) to elucidate community assembly processes and evaluate key factors influencing microbial dynamics. The results indicate that microbial community assembly does not vary linearly with reef distance; rather, lower stochastic processes and species dispersal levels occurred at 0 m and 3 m (1.5 times reef height). The flow-field effects generated by AR units significantly influenced microbial spatial distribution, primarily by altering hydrodynamics and thus affecting sediment particle deposition and nutrient transport [32]. Due to the obstruction of water flow by reefs, an upwelling zone forms in front of the reef, while an eddy zone develops behind it, both reducing local water velocities but differing in extent and intensity of impact (Figure 9). In front of the reef, currents deflect sharply upward, partially accelerating laterally and partially downward, causing sediment erosion near the reef [68]. Conversely, the rear eddy zone connects upper and lower water layers, characterized by lower velocities but enriched nutrients, thus providing favorable conditions for microbial growth and metabolism [13].

Laoshan Bay is typically characterized by reciprocating tidal flows, and sampling was conducted along the flow direction. When the water flow direction aligned with the sampling direction, the microbial community at 0 m was influenced by low velocity conditions due to reef blockage; when reversed, the community was influenced by the reef-induced eddy. Consequently, the lower variance explained and species dispersal observed at 0 m may result from the combined effects of upwelling and eddy flows. While microbial communities at 1 m, 3 m, and 5 m were all influenced by the rear eddy zone, communities at 3 m showed the lowest Nm values, indicating restricted species dispersal possibly due to proximity to the eddy center. In contrast, the higher variance explained and Nm at 5 m suggest stronger influences from stochastic processes, such as dispersal and ecological drift, reflecting weaker reef-driven flow effects. Hence, AR unit hydrodynamic effects modify not only sediment deposition patterns but also microbial dispersal and assembly mechanisms, ultimately shaping the spatial distribution and ecological functions of sediment microbial communities.

### 4.4. Environmental Drivers and Ecological Regulation of Microbial Communities

This study reveals that the sediment microbial community structure surrounding AR units is dynamically regulated by environmental factors, with significant differences in key drivers observed across reef distances. Overall, available sulfur (AS), organic matter (OM), water content (WC), pH, and ammonium nitrogen (NH_4_^+^-N) significantly influenced microbial community structure at varying reef distances, although their relative contributions differed spatially. Specifically, AS consistently exhibited a high and stable contribution across distances, indicating its enduring role in shaping microbial community structure [70]. In contrast, OM showed the highest contributions at distances of 0 m and 3 m, whereas pH and NH_4_^+^-N peaked at 3 m. WC exerted the greatest influence at the 0 m site. These patterns indicate that AR units shape distinct microbial niches at various distances, driving microbial spatial differentiation through modifications in hydrodynamic conditions, nutrient cycling, and sediment physicochemical properties.

Correlation analysis between dominant microbial phyla and environmental factors further supported these findings. Organic matter (OM) showed no significant influence at distances of 0 m and 5 m from the reef, but exhibited strong positive correlations with several dominant phyla at 1 m and 3 m. This suggests that OM may influence microbial community composition and metabolic activity at specific distances by modulating carbon source availability. The enrichment of phyla such as Acidobacteriota, Chloroflexota, Verrucomicrobiota, Latescibacterota, and Sumerlaeota likely enhances the degradation of sedimentary organic matter, thereby promoting carbon cycling [42,71,72,73,74]. Additionally, pH regulation varied spatially; at distances of 0 m and 1 m, pH correlated positively with some dominant phyla, whereas at 3 m and 5 m, negative correlations were observed. Those suggesting that pH may influence microbial community structure and function by altering sediment geochemistry and modulating ecological niche competition. Notably, elevated pH values at 3 m and 5 m appeared to suppress the growth of several anaerobic phyla, including Spirochaetota, Deferrisomatota, Thermodesulfobacteriota, Chloroflexota, and Methylomirabilota, all of which are closely associated with anaerobic metabolic processes [75,76,77,78]. This inhibition also affected key biogeochemical cycles, particularly sulfur cycling (e.g., Thermodesulfobacteriota) and nitrogen cycling (e.g., Nitrospirota and Bacillota) [35,79,80]. Furthermore, sediment physical parameters (WC and BD) displayed distinct spatial relationships with microbial communities. Higher WC typically facilitates oxygen and nutrient transport, affecting sediment porosity and microbial habitat availability [81]. Collectively, these findings suggest that AR units shape distinct sediment microbial habitats at different distances, driving microbial succession and spatial differentiation through combined hydrodynamic, nutrient, and physicochemical regulatory processes.

## 5. Conclusions

This study systematically examined the spatial and seasonal dynamics of sediment microbial communities at different reef distances in Laoshan Bay. Results demonstrate that the spatial distribution of dominant microbial phyla around AR units was influenced jointly by season, reef distance, and reef characteristics, promoting the enrichment of functional microbial groups such as carbohydrate-degrading, sulfur-cycling, and nitrogen-cycling taxa. Significant spatial differences in microbial community structure were observed across reef distances, notably at 3 m (1.5 times reef height). Although the spatial enrichment patterns of distinct microbial taxa varied seasonally, microbial ecological traits (aerobic or anaerobic) remained stable. This indicates that AR units create favorable local environments for different microbial types without altering their fundamental ecological characteristics. Furthermore, the Neutral Community Model (NCM) revealed lower stochastic processes and dispersal at distances of 0 m and 3 m, which are relatively consistent with the flow field effects (upwelling and reverse eddy currents). Key environmental factors and their regulatory roles differed markedly with reef distances. AR units thus shape spatially distinct microbial habitats, driving microbial succession and differentiation via hydrodynamic modulation, nutrient supply, and physicochemical adjustments. This study highlights the complex regulatory mechanisms of AR units on sediment microbial communities, emphasizing spatio-temporal heterogeneity influenced by both physical conditions and biological interactions. These insights provide valuable theoretical support for artificial reef planning, ecological evaluation, and optimized management, contributing significantly to marine ecological restoration and sustainable fishery resource utilization.

## Data Availability

The original contributions presented in this study are included in the article. Further inquiries can be directed to the corresponding author.

## Appendix A

### Soil Physicochemical Analysis

Soil physicochemical properties, including nitrate-N (NO_3_^−^-N), ammonium-N (NH_4_^+^-N), available sulfur (AS), total phosphorus (TP), inorganic phosphorus (IP), and organic phosphorus (OP), were quantified using corresponding detection kits (Solarbio, Beijing, China). These kits include a series of reagents prepared by the manufacturer based on the detection principles of specific physicochemical indicators. Standard reagents were used for calibration before each measurement.

According to the instructions provided with the test kits, soil NO_3_^−^-N and NH_4_^+^-N were determined using salicylic acid colorimetry, (1-naphthyl)-diaminoethane spectrophotometry, and indophenol blue spectrophotometry methods [82]. For NO_3_^−^-N analysis, soil samples were extracted with distilled water (1:10, *w*/*v*) for 1 h and centrifuged. Under alkaline conditions (pH > 12), NO_3_^−^-N reacts with salicylic acid to form nitrosalicylic acid, which turns yellow. The absorbance was measured at 410 nm using kit BC0040 (Solarbio), with a detection limit of 0.0495 μg/g [83]. NH_4_^+^-N was determined using indophenol blue spectrophotometry. Soil was extracted with 2 mol/L KCl solution (1:10, *w*/*v*) for 1 h, followed by centrifugation. Under strong alkaline conditions, NH_4_^+^-N reacts with hypochlorite and phenol to form indophenol blue dye. The absorbance was measured at 630 nm using kit BC1510 (Solarbio), with a detection limit of 0.0646 μg/g [84]. Phosphorus content was determined colorimetrically using the molybdate blue method [85]. IP was measured via direct extraction. OP was determined by calculating the difference between TP and IP, where TP was measured after high-temperature ashing, converting organic phosphorus to inorganic phosphorus. Phosphates react with ammonium molybdate to form pale yellow phosphomolybdate, which is then reduced by stannous chloride to form blue-colored molybdenum blue. The absorbance was measured at 660 nm using kit BC2890 (Solarbio), with a detection limit of 0.18 μg/g. Available sulfur (AS) was measured using the BaSO_4_ turbidimetric method. Soil sulfur was extracted using a CaCl_2_ solution, and the turbidity of the BaSO_4_ formed was measured at 440 nm with kit BC2980 (Solarbio), yielding a detection limit of 2.8927 mg/kg [86].

**Table A1:** Comparison of the bacterial relative abundance (phylum) around the AR units (reef–reef distance) in different seasons.

Season	Reef–Reef Distance	*Acidobacteriota*	*Thermodesulfobacteriota*	*Actinomycetota*	*Bacteroidota*	*Chloroflexota*	*Myxococcota*	*Nitrospirota*	*Bacillota*	*Verrucomicrobiota*
May	R 1-0	11.33 b	13.59 a		5.15 a	5.05 b				1.88 b
	R 1-1	7.63 a	15.36 ab		7.55 ab	3.76 ab				0.97 ab
	R 1-3	7.41 a	16.41 b		8.99 ab	3.23 ab				1.2 ab
	R 1-5	6.77 a	15.99 ab		10.04 b	2.77 a				0.88 a
	R 2-0	8.03 c							1.12 b	
	R 2-1	5.54 a							0.91 ab	
	R 2-3	6.43 b							0.77 ab	
	R 2-5	4.95 a							0.56 a	
	R 3-0	5.91 a	7.2 a	9.82 b			2.5 a		2.91 b	
	R 3-1	6.87 b	7.85 a	7.34 ab			2.88 a		1.17 a	
	R 3-3	6.54 ab	7.53 a	5.35 a			3.42 b		0.51 a	
	R 3-5	9.67 c	11.06 b	6.53 ab			2.96 ab		1.39 a	
August	R 1-0	8.46 a	7 a			1.89 a	2.51 a	2.78 b		
	R 1-1	7.99 a	7.32 a			2.27 ab	2.5 a	1.87 ab		
	R 1-3	11.02 b	17.64 b			3.44 b	4.89 b	1.68 a		
	R 1-5	9.17 a	9.1 a			2.68 ab	3.5 a	1.74 ab		
	R 2-0	11.63 b	14.41 a					2.71 b		
	R 2-1	10.75 ab	15.77 ab					2.56 b		
	R 2-3	9.67 a	11.99 a					1.96 a		
	R 2-5	9.72 a	19.11 b					2.57 b		
February	R 1-0	10.1 c	14.3 b	6.32 a	10.56 a		5.6 b		1.63 b	1.66 a
	R 1-1	8.75 bc	12 b	7.24 ab	9.89 a		3.66 ab		1.04 a	2.85 ab
	R 1-3	7.57 ab	7.84 a	9.26 b	15.65 ab		3.11 a		1.02 a	6.35 ab
	R 1-5	6.09 a	7.74 a	6.01 a	21.04 b		2.46 a		0.82 a	7.39 b
	R 2-0		3.92 a	10.12 b				0.64 a		
	R 2-1		5.02 ab	7.41 a				0.99 ab		
	R 2-3		5.39 b	9.98 ab				1.25 b		
	R 2-5		6.24 b	8.39 ab				1.16 b		
	R 3-0			5.28 a						
	R 3-1			6.95 b						
	R 3-3			7.08 bc						
	R 3-5			8.26 c						

Note: Means with the same letter are not significantly different, while those with different letters indicate a significant difference (*p* < 0.05).

**Table A2:** Sediment environmental factors at 0 m, 1 m, 3 m and 5 m away from the reef in different seasons. OM—organic matter content; BD—sediment bulk density; WC—water content; NH_4_^+^-N—ammonium-N; NO_3_^−^N—nitrate-N; AS—available sulfur; IP—inorganic phosphorus; TP—total phosphorus; OP—organic phosphorus.

Season	Reef Distance	OM (%)	pH	BD (g/cm^3^)	WC (%)	NH_4_^+^-N (μg/g)	NO_3_^−^-N (μg/g)	AS (mg/kg)	IP (μg/g)	TP (μg/g)	OP (μg/g)
May	R_0	5.6 ± 2.4	9.12 ± 0.35	0.08 ± 0.02	41.16 ± 11.7	4.25 ± 0.46	1.29 ± 1.11	25.61 ± 26.72	157.09 ± 140.15	219.93 ± 190.59	62.84 ± 67.77
	R_1	5.65 ± 1.8	9.13 ± 0.28	0.08 ± 0.02	53.02 ± 13.92	5.19 ± 0.21	0 ± 0	38.05 ± 33.06	134.65 ± 123.41	201.98 ± 181.16	67.33 ± 58.69
	R_3	6.54 ± 0.83	9.22 ± 0.35	0.09 ± 0.02	51.07 ± 16.51	4.28 ± 0.33	1.93 ± 3.34	40.37 ± 38.74	161.58 ± 139.93	273.79 ± 237.97	112.21 ± 99.25
	R_5	6.7 ± 1.57	9.19 ± 0.31	0.08 ± 0.02	53.44 ± 18.99	4.31 ± 0.95	5.15 ± 7.31	33.4 ± 31.59	152.61 ± 134.88	255.84 ± 222.48	103.23 ± 101.06
August	R_0	6.94 ± 1.4	9.2 ± 0.05	0.09 ± 0.02	42.91 ± 10.28	2.82 ± 2.45	5.15 ± 4.86	64.63 ± 11.39	121.19 ± 105.17	350.1 ± 303.49	228.91 ± 199.27
	R_1	6.47 ± 0.32	9.2 ± 0.42	0.08 ± 0.03	67.03 ± 31.33	2.8 ± 2.44	9.65 ± 12.06	54.99 ± 26.9	116.7 ± 104.59	341.12 ± 295.42	224.42 ± 196.21
	R_3	5.92 ± 1.85	8.9 ± 0.13	0.09 ± 0	53.2 ± 4.05	3.68 ± 3.25	12.23 ± 15	85.64 ± 56.02	143.63 ± 125.11	345.61 ± 299.38	201.98 ± 175.05
	R_5	6.12 ± 1.35	9.24 ± 0.28	0.07 ± 0.02	54.01 ± 1.89	2.84 ± 2.46	4.5 ± 4.02	67.13 ± 18.61	134.65 ± 117.39	336.63 ± 293.47	201.98 ± 181.16
November	R_0	6.35 ± 1.72	8.98 ± 0.41	0.07 ± 0.02	78.41 ± 43.64	3.78 ± 0.69	5.15 ± 2.95	51.83 ± 17.51	210.96 ± 77.74	287.26 ± 7.77	76.3 ± 74.16
	R_1	7.61 ± 0.87	9.05 ± 0.23	0.08 ± 0.02	68.21 ± 29.85	4.91 ± 1.53	7.08 ± 8.92	58.26 ± 7.87	242.37 ± 23.32	287.26 ± 7.77	44.88 ± 28.03
	R_3	6.22 ± 2.35	9.24 ± 0.12	0.07 ± 0.01	45.22 ± 14.82	4.03 ± 1	3.22 ± 1.11	44.81 ± 9.24	193 ± 50.98	318.68 ± 15.55	125.68 ± 66.42
	R_5	6.59 ± 1.71	9.22 ± 0.3	0.07 ± 0.02	62.72 ± 11.15	3.56 ± 0.24	3.22 ± 1.11	56 ± 34.26	201.98 ± 35.63	377.03 ± 61.71	175.05 ± 81.91
February	R_0	10.01 ± 6.46	9.12 ± 0.21	0.09 ± 0.01	44.88 ± 5.5	4.03 ± 0.03	2.57 ± 2.23	34.81 ± 2.75	228.91 ± 48.55	327.65 ± 56.06	98.74 ± 89.66
	R_1	12.09 ± 8.48	9.07 ± 0.09	0.09 ± 0	51.28 ± 6.03	3.89 ± 0.26	1.29 ± 1.11	53.5 ± 21.01	233.4 ± 47.29	323.17 ± 110.22	89.77 ± 89.66
	R_3	11.55 ± 3.8	9.14 ± 0.05	0.09 ± 0.01	49.6 ± 1.18	3.99 ± 0.29	1.93 ± 0	61.71 ± 35.59	237.89 ± 41.14	336.63 ± 40.4	98.74 ± 81.16
	R_5	7.45 ± 0.73	8.89 ± 0.04	0.08 ± 0.01	67.16 ± 11.89	3.68 ± 0.64	7.72 ± 3.34	31.48 ± 11.58	206.47 ± 28.03	363.56 ± 58.69	157.09 ± 60.72
